# The Association Between Health Literacy and General Health in Women With Gestational Diabetes: A Cross-sectional Study

**DOI:** 10.34172/jrhs.11438

**Published:** 2025-10-18

**Authors:** Yasaman Rajabi Basir, Sara Alipour, Farzaneh Esna-Ashari, Shiva Borzouei

**Affiliations:** ^1^Department of Medicine, School of Medicine, Hamadan University of Medical Sciences, Hamadan, Iran; ^2^Research Center for Social Factors Affecting Health, Hamadan University of Medical Sciences, Hamadan, Iran; ^3^Department of Endocrinology, School of Medicine, Hamadan University of Medical Sciences, Hamadan, Iran

**Keywords:** Gestational diabetes, Health literacy, General health

## Abstract

**Background:**

Health literacy includes cognitive and social skills that enable individuals to understand and use health information effectively. In addition, it significantly influences health outcomes in society. Women with gestational diabetes mellitus (GDM) often have low health literacy and need better education. Therefore, this study explored the link between health literacy and general health in these women.

**Study Design:**

A cross-sectional study.

**Methods:**

This study involved 200 women with GDM referred to the Diabetes Clinic in Hamadan, Iran. The participants were selected through consecutive sampling, and the required data were collected using self-reported questionnaires, a health literacy questionnaire, and a general health questionnaire. The obtained data were analyzed using SPSS with a 95% confidence level.

**Results:**

The mean age of women was 29.63 years, and the mean±standard deviation (SD) of health literacy score was 77.41±16.44. Further, the mean±SD of the general health questionnaire score was 21.02±6.01. There was a positive correlation between health literacy and general health (*P*<0.001), as well as between health literacy and education (*P*<0.05). Moreover, a positive correlation was found between general health and education (*P*<0.05). Eventually, a significant negative correlation was observed between age and general health (*P*<0.05).

**Conclusion:**

Health literacy plays an essential role in managing GD and promoting general health for pregnant women. This subsequently leads to reduced postpartum complications for the mother and baby, as well as reduced healthcare costs.

## Background

 The responsibility of individuals toward health and self-care has considerably increased in both developed and developing countries. In addition, access to health-related information and awareness of health issues are crucial determinants of overall health.^1^ With recent improvements in health status and care, individuals face new health-related needs that require them to make informed decisions.^2^

 Health literacy refers to social and cognitive skills that enable people to access, understand, and use health information effectively in order to promote and maintain good health. It reflects a person’s ability to obtain, interpret, and comprehend the information required for health-related services and to make informed decisions regarding their health.^3^ Health literacy is a crucial factor when determining healthcare outcomes and costs, making it essential for the healthcare system to promote high health literacy.^4^

 Research indicates that individuals with low health literacy often struggle to understand both written and spoken information from healthcare professionals. This difficulty can lead to poor adherence to medical instructions and negatively impact their overall health. As a result, they tend to experience higher hospitalization rates and have less effective self-care skills.^5^

 Health literacy is crucial during pregnancy, as a mother’s health behaviors directly impact both her own well-being and that of her baby. According to a systematic review, the levels of health literacy among pregnant women varied significantly across different studies, with most findings being unfavorable. These varying levels of health literacy are reported to influence the health of pregnant women and the outcomes of their pregnancies. Research on health literacy indicates that inadequate health literacy is associated with smoking habits, heightened risk perceptions, negative beliefs about medications, and non-adherence to prescribed treatments, which all affect pregnant women.^6^

 Gestational diabetes mellitus (GDM) is characterized by glucose intolerance that starts or is first identified during pregnancy. It is one of the most prevalent metabolic disorders that occurs during pregnancy^7,8^ and a significant contributor to negative perinatal outcomes.^9^ A significant part of GDM management involves educating pregnant women about diet, exercise, self-management, and insulin monitoring to enhance maternal and fetal outcomes.^10^ Increasing diabetes knowledge is related to improved functional, communicative, and critical health literacy. Further, individuals with adequate disease knowledge tend to communicate more confidently and comfortably with healthcare professionals.^11^

 Despite the high prevalence of GDM and the vital importance of health literacy in managing GDM, the evaluation of health literacy status among pregnant women is not sufficiently incorporated into standard antenatal services.^12^ According to some studies, inadequate health literacy levels are a widespread issue, particularly for women with GDM, who require better and more effective education.^12,13^

 Additionally, women with GDM tend to demonstrate a higher prevalence of mental health disorder symptoms, which may be linked to a less healthy lifestyle. Depressed women with GDM often reduce their use of social support and experience significant concerns about their condition and treatment. This situation can lead to increased depression, thereby creating a vicious cycle that further diminishes their quality of life.^14^ Given the absence of research exploring the relationship between health literacy and general health scores in pregnant women with GDM, an indicator of general health, this study has been designed to examine how health literacy impacts the general health scores of these women.

## Methods

###  Study design and participants

 This cross-sectional study was conducted on 200 pregnant women with a diagnosis of GDM who presented to the diabetes clinic in Hamadan Province from 2022 to 2023.

 The inclusion criteria for participation in the research were having GDM, possessing basic literacy skills, showing a willingness to take part in the study, and completing consent forms.

 On the other hand, the exclusion criteria included pregnant women with overt diabetes, pregnant women who were ill, blind, or deaf, individuals with cognitive impairments or communication difficulties, and those who were unwilling to participate in the study.

###  Sampling

 The samples were selected using convenience sampling from the Specialized Endocrinology Clinic in Hamadan province from 2022 to 2023. The sample size was calculated as 200 people based on a confidence interval of 95%, a test power of 80%, and an effect size of 0.2.^15^

###  Instruments

 The data collection tools included a researcher-made questionnaire to capture patients’ demographic characteristics, such as age, education, and place of residence, as well as their pregnancy history, which comprised the number of pregnancies, the nature of any unwanted pregnancies, and the source of health information. A health literacy questionnaire and a general health questionnaire were also used for data collection.

 The Health Literacy Questionnaire, developed by Montazeri et al (2014), contains 33 items divided into five components: accessibility (items 1–6), reading skills (items 7–10), comprehension (items 11–17), evaluation (items 18–21), and decision-making and application of health information (items 22–33). The reliability of the items within the relevant constructs, measured by Cronbach’s alpha, ranged from 0.72 to 0.89, confirming that the questionnaire is reliable. In addition, in the study of Montazeri its validity was assessed using exploratory factor analysis, and the Kaiser–Meyer–Olkin value was 0.919 at a significant level of *P* < 0.001.^16^

 All items were scored using a Likert-type scale ranging from 1 to 5 (1 = never, 2 = rarely, 3 = sometimes, 4 = usually, and 5 = always). The score for each domain was separately calculated, resulting in an overall score for all combined domains. In this study, health literacy was categorized into “inadequate” (0–50), “not adequately sufficient” (50.1–66), “sufficient” (66.1–84), and “excellent” (84.1–100) levels. The raw score for each domain was obtained by summing the scores of each item’s responses. Further calculations were applied to convert this raw score into a percentage in the range of 0–100.

 The standard general health questionnaire (Iranian version) has been scored and validated, achieving a reliability coefficient of 0.87. This 12-item General Health Questionnaire was developed as a tool to assess general health. Each item is rated on a four-point scale: “less than usual”, “no more than usual”, “rather more than usual”, or “much more than usual”. For instance, when using the GHQ-12, the total score can be either 36 or 12, depending on the selected scoring method. The most common scoring methods are bi-modal (0-0-1-1) and Likert scoring styles (0-1-2-3). The higher the score on this questionnaire, the lower the level of general health, and vice versa.^17^

###  Data analysis

 The obtained data were statistically analyzed using SPSS, version 26. Descriptive data were presented in graphs and tables, including frequencies, means, and standard deviations (SD). In the analytical section, the normality of the data was evaluated using the Kolmogorov-Smirnov test to compare the mean and SD of health literacy and general health scores across various independent and contextual variables. Based on the normality of the data distribution, the Pearson correlation coefficient and the Student’s t-test were employed for data analysis and group comparisons. A *P*-value of less than 0.05 was considered statistically significant.

## Results

###  Sociodemographic characteristics

 The mean age of the participants was 29.63 years with an SD of 5.36 years, ranging from 17 years to 43 years. Further, the average number of pregnancies was 2.25, with an SD of 0.72 and a range of 1–6. One hundred three (51.5%) women resided in urban areas, while ninety-seven (48.5%) lived in rural areas. According to the findings, 19.5% of the participants (n = 39) had secondary and high school education, while 80.5% of them (n = 161) had university education. Out of 200 pregnant women, 151 (75.5%) desired the pregnancy, while 49 (24.5%) experienced an unwanted pregnancy.

###  Health literacy

 The mean ± SD of health literacy scores for women participating in the study was 14.05 ± 3.55, 9.33 ± 2.71, 16.37 ± 4.26, and 9.54 ± 2.62 for reading proficiency, understanding, assessment, and decision making and information application, respectively, and the overall score was 77.41 ± 16.44. Table 1 presents the mean of the total score and subdomains of health literacy among pregnant women participating in the study.

**Table 1:** The mean of the total score and subdomains of health literacy among pregnant women

**Variables**	**Minimum**	**Maximum**	**Mean**	**SD**
Access	6	30	14.05	3.55
Reading	4	20	9.33	2.71
Understanding	7	35	16.37	4.26
Evaluation	4	20	9.54	2.62
Decision-making	12	60	28.11	6.44
Total	33	165	77.41	16.44

*Note*. SD: Standard deviation.

###  General health

 The mean ± SD of the general health score was 21.02 ± 6.01. It is important to note that in the general health questionnaire, a higher score indicates a worse state of health.

###  Relationships 

 The comparison results of the mean scores for health literacy and general health based on participants’ residence, education, and pregnancy tendencies using the t-test indicated that both health literacy and general health were higher among women with higher education. This difference was statistically significant, with *P*-values of 0.037 and 0.012, respectively. Moreover, women with intended pregnancies demonstrated insignificantly better general health (*P* = 0.120) compared to those with unwanted pregnancies. Although they also showed higher health literacy, this difference was not significant (*P* = 0.399). Likewise, health literacy and general health levels were superior in women living in cities, but the differences in both levels were not significant (*P* = 0.159 and 0.321, respectively). Table 2 lists the mean and SD of general health and health literacy scores based on the participants’ residence, education, and pregnancy tendencies.

**Table 2:** The mean of general health and health literacy scores based on the participants’ residence, education, and pregnancy tendencies

**Variables**	**Health literacy score**			**General health score**		
	**Mean**	**SD**	***P *** **value**	**Mean**	**SD**	***P *** **value**
Residence			0.159			0.321
Urban	79.00	15.46		20.65	5.87	
Rural	75.70	17.53		21.49	6.12	
Education			0.037			0.012
Secondary and high school	72.51	18.49		23.15	6.23	
University	79.22	15.54		20.55	5.89	
Tendency to pregnancy			0.399			0.12
Intended	77.91	15.81		19.93	5.85	
Unintended	75.63	17.01		20.88	6.25	

*Note*. SD: Standard deviation.

###  Correlations 

 There was a significant negative correlation between age and general health, indicating that older women had better general health (r = -0.255, *P* < 0.001). Additionally, a weak correlation was observed between age and health literacy, suggesting that older women may have higher health literacy; however, this finding was not statistically significant (r = 0.123, *P* = 0.166). The number of pregnancies did not have a meaningful effect on either health literacy scores or general health (*P*-values of 0.598 and 0.447 and correlation coefficients of r = 0.123 and r = 0.151, respectively). Furthermore, a significant negative correlation was found between general health and health literacy according to the Pearson correlation coefficient (r = -0.531, *P* < 0.001), demonstrating that lower health literacy is associated with poorer general health. Table 3 provides the correlations between health literacy scores, general health, age, and the number of pregnancies.

**Table 3:** Correlation between health literacy score, general health, age, and number of pregnancies

**Variables**	**Health literacy score**		**General health score**	
	***P *** **value**	**r**	***P *** **value**	**r**
Age (year)	0.166	0.123	0.001	-0.255
Number of pregnancies	0.598	0.055	0.447	0.151
General health	0.001	-0.531	1.000	1.000

## Discussion

 The mean health literacy score was 77.41, representing that, on average, most women achieved at least an “adequate” level. However, a significant proportion of women remain below the desired range. The mean general health score of 21.02 indicates a low general health score, highlighting the need for targeted interventions.

 The result of this study revealed the suboptimal level of health literacy among women with GDM. The results of a systematic review conducted by Nawabi et al on health literacy among pregnant women confirmed that pregnant women, particularly in Western high-income countries, exhibited an adequate level of health literacy.^6^

 A study examining the electronic health literacy of pregnant women with GDM reported that their health literacy levels are suboptimal and need improvement. The sources from which these women access health information significantly influence their electronic health literacy. Additionally, a lack of knowledge about how to obtain information from electronic devices can contribute to low health literacy among women with GDM.^18^ These results are in line with those of our study, demonstrating that women who received information from physicians, healthcare staff, and educational/promotional booklets and pamphlets had a different experience compared to those who obtained information from other sources. This difference may be attributed to a lack of literacy regarding how to access information from electronic devices.

 In assessing general health using a general health questionnaire, the results indicated a suboptimal level of general health among women with GDM participating in our study. Similarly, Grinberg and Yisaschar-Mekuzas examined variations in the levels of anxiety, depression, stress, and somatization among women with GDM compared to healthy pregnant women and explored differences based on diabetes control.^19^ Lee et al found that 40% of women with GDM experienced anxiety, while 10% also exhibited symptoms of depression and stress.^20^ These findings align with those of our research.

 Based on the findings of another study, a history of depression before pregnancy could increase the risk of GDM.^21^ It should be clarified whether poor general health contributes to GDM or if GDM can cause general health disorders.

 There was a significant negative correlation between general health and health literacy among the studied women, implying that lower health literacy is associated with poorer general health.

 Seyedoshohadaee et al, investigating the relationship between health literacy and general health in patients with type 2 diabetes, concluded that individuals with this condition experience various clinical, social, and psychological challenges. According to them, these challenges often lead to both mental and physical limitations. They further reported that inadequate health literacy and public health issues are prevalent among diabetic patients, emphasizing the need for better education to enhance their health literacy and overall health.^15^

 This association has substantial implications, as inadequate health literacy may undermine women’s ability to understand medical instructions, perform effective self-management, and access relevant support services, ultimately resulting in compromised mental and physical health. Considering that women with GDM are already at higher risk of psychological disorders (e.g., depression and anxiety), poor health literacy may exacerbate these issues, thus perpetuating a cycle of poor health that affects both the mother and, potentially, her child. The direction and strength of these correlations emphasize that healthcare systems should prioritize the assessment and enhancement of health literacy as integral parts of antenatal services.^22,23^

 Our results confirmed a significant negative correlation between age and general health, indicating that older women experienced better general health. In contrast to our study, other research examining general health disorders following GDM showed that the general health of women with GDM deteriorated with age.^24^ Age itself cannot be a predictor of the severity of general health disorders, as it can be influenced by other factors, such as ethnicity and culture.

 In the present study, no significant relationship was observed between health literacy scores and general health with the number of pregnancies and births, place of residence, and desired and undesired pregnancies. However, a significant relationship was found between higher education levels and improved health literacy and general health. It is noteworthy that reading literacy is the foundation of health literacy. When patients have adequate literacy skills, they can better understand the educational materials provided by healthcare professionals. Additionally, strong literacy skills help individuals engage with a variety of printed, visual, and auditory educational resources, which enable them to take advantage of different forms of communication, including electronic social networks.^25^ Research conducted over the past 20 years has shown that adults with low literacy typically have less understanding of health issues, struggle to manage their conditions, are less likely to access preventive health services, and have a higher chance of being hospitalized.^26^

 This study had some limitations. The cross-sectional design of this study prevented establishing causal relationships between variables and the use of self-report questionnaires, which may introduce response bias. Furthermore, the sample, although representative of the local population, may have limited the ability to generalize the findings internationally or to settings with greater diversity in socioeconomic or educational backgrounds. Hence, future research using longitudinal designs and including intervention arms can further elucidate the impact of structured educational programs delivered by healthcare professionals on health literacy and subsequent clinical outcomes in GDM.

**Highlights**

- Women with gestational diabetes have poor general health.
- These women have adequate levels of health literacy.
- Lower health literacy is associated with poorer general health.

## Conclusion

 It was revealed that health literacy plays a critical role in managing GDM and improving general health outcomes for pregnant women. Based on the findings, adequate health literacy enables women with GDM to understand their condition and be more compliant with their doctors’ orders, including dietary recommendations, medication instructions, and regular blood sugar monitoring. It was further found that pregnant women with GDM are better able to prevent complications for themselves and their babies when they have high health literacy. This, in turn, contributes to a healthier pregnancy and postpartum period and reduces the burden on public health systems. Conversely, low health literacy could lead to poor disease management, increased risk of complications, and higher healthcare costs.

## Acknowledgements

 This study was approved by the Research Vice-Chancellor of Hamadan University of Medical Sciences (1401120210373). The authors would like to express their sincere thanks.

## Competing Interests

 The authors have no conflict of interests.

## Ethical Approval

 This study was conducted in accordance with the principles of the Helsinki Declaration and was approved by the Ethics Committee of Hamadan University of Medical Sciences (IR.UMSHA. REC.1401.951).

## Funding

 This research received no specific grant from funding agencies in the public, commercial, or not-for-profit sectors.
